# Machine Learning to Distinguish Physiologic Causes of Hypoxemia After Bidirectional Glenn Using Bedside Markers

**DOI:** 10.3390/children13101414

**Published:** 2026-10-15

**Authors:** Fabio Savorgnan, Pranathi Pilla, Sarah Visokay, Kaitlin Ness, Thomas Fiedorek, Saul Flores

**Affiliations:** 1 Department of Pediatrics, Division of Critical Care, Baylor College of Medicine, Houston, TX 77030, USA; tjfiedor@texaschildrens.org (T.F.); saul.flores2@bcm.edu (S.F.); 2 Baylor College of Medicine, Houston, TX 77030, USA; pranathi.pilla@bcm.edu (P.P.); sarah.visokay@bcm.edu (S.V.);

**Keywords:** bidirectional Glenn, hypoxemia, machine learning, bedside monitoring, central venous pressure, venous oxygen saturation, single ventricle

## Abstract

**Highlights:**

**What are the main findings?**

- In this mechanistic simulation, SpO_2_ alone provided limited recovery of the encoded physiological state, whereas Glenn/SVC pressure and SVC oxygen saturation provided the largest incremental information.
- Elevated pulmonary vascular resistance was the most recoverable encoded state, while veno-venous collateral shunting and other mechanisms showed substantial overlap; performance deteriorated as measurement uncertainty increased.

**What are the implications of the main findings?**

- The findings are model-dependent and hypothesis-generating and should not be interpreted as clinical diagnostic accuracy.
- Prospective clinical validation should test whether pressure and venous oxygenation measurements add useful information to SpO_2_ in real Glenn patients.

**Abstract:**

Background: Hypoxemia after bidirectional Glenn palliation can result from distinct physiological mechanisms that may produce similar arterial oxygen saturation. We evaluated whether routinely available bedside markers could recover physiological states encoded in a mechanistic virtual-patient model. Methods: A steady-state zero-dimensional closed-loop model generated five causes of Glenn hypoxemia: elevated pulmonary vascular resistance, veno-venous collateral shunting, pulmonary gas-exchange impairment, increased metabolic demand, and ventricular dysfunction. Hidden physiological variables were withheld from the classifier; inputs were SpO_2_, heart rate, mean arterial pressure, hemoglobin, Glenn/SVC pressure, and SVC oxygen saturation. Because bedside patterns arise downstream from prespecified equations and phenotype perturbations, classification was interpreted as internal mechanistic recoverability rather than clinical diagnostic performance. Results: Of 150,000 generated states, 127,162 passed broad physiological criteria and 66,269 met strict hypoxemia/hemodynamic criteria. After balancing, 26,400 virtual patients were analyzed (5280 per mechanism; 19,800 training and 6600 held-out testing). SpO_2_ alone achieved 26.1% accuracy (macro-AUROC 0.580); the primary bedside model achieved 56.1% accuracy (95% CI 55.0–57.4%) and macro-AUROC 0.845 (95% CI 0.840–0.851). Elevated PVR was the most readily recovered phenotype. Glenn/SVC pressure and SVC saturation provided the largest incremental contributions, while broader measurement-error assumptions substantially reduced performance. Conclusions: Within this mechanistic simulation, multivariable bedside patterns allowed partial recovery of physiological states encoded by the model. Because phenotypes and their observable consequences were generated from prespecified assumptions, the reported performance quantifies internal recoverability under those assumptions and should not be interpreted as diagnostic accuracy or as independent evidence of bedside information content in patients. The model identifies testable physiologic signatures and areas of overlap for prospective clinical validation.

## 1. Introduction

The bidirectional Glenn is an established stage of single-ventricle palliation [1]. Hypoxemia is common in patients with bidirectional Glenn physiology, but arterial oxygen saturation alone provides limited information about the mechanism producing it. Following the Glenn, superior vena caval blood reaches the pulmonary arteries without a subpulmonary ventricle, making pulmonary blood flow dependent on systemic venous return, the transpulmonary pressure gradient, pulmonary vascular resistance, and downstream atrial pressure [2,3,4]. Consequently, similar systemic oxygen saturations may occur in markedly different hemodynamic states.

Several mechanisms can contribute to Glenn hypoxemia. Increased pulmonary vascular resistance may impair passive pulmonary blood flow and increase Glenn pressure [3,4,5], whereas veno-venous collaterals permit systemic venous blood to bypass effective pulmonary oxygenation and may decompress the superior caval circulation [6,7]. Pulmonary gas-exchange abnormalities can reduce systemic saturation without a corresponding reduction in pulmonary blood flow; in Glenn patients, supplemental oxygen has been shown to increase arterial saturation without materially changing Qp/Qs [8]. Increased metabolic demand and reduced systemic output may further lower venous oxygen saturation through increased extraction or impaired oxygen delivery [2,9,10]. Importantly, reduced cardiac output need not initially produce hypotension: compensatory vasoconstriction can preserve arterial pressure despite falling output, and hypotension may therefore be a later sign of low-output physiology in infants [11]. These mechanisms may consequently produce similar cyanosis while generating different pressure, flow, and venous oxygenation profiles.

At the bedside, however, these underlying physiological disturbances are not directly measured. Clinicians instead integrate readily available variables such as SpO_2_, systemic arterial pressure, Glenn or central venous pressure, heart rate, hemoglobin concentration, and superior vena caval or central venous oxygen saturation. Clinical observations support the value of considering these signals together. During transient obstruction of the cavopulmonary pathway, increased central venous pressure has been accompanied by decreases in SpO_2_, central venous oxygen saturation, and cerebral near-infrared spectroscopy [12]. Similarly, pulmonary vasodilation with inhaled nitric oxide after Glenn has been associated with lower central venous pressure and improved arterial and venous oxygen saturation [5]. Whether combinations of these routinely available bedside measurements contain sufficient information to distinguish among major mechanisms of Glenn hypoxemia has not been systematically evaluated.

We therefore developed a physiology-based virtual-patient model in which the underlying cause of hypoxemia was known but withheld from the classifier. We tested whether machine learning using only routinely available bedside markers could distinguish five major physiologic mechanisms: elevated pulmonary vascular resistance, veno-venous collateral shunting, pulmonary gas-exchange impairment, increased metabolic demand, and ventricular dysfunction. We hypothesized that arterial oxygen saturation alone would provide limited mechanistic discrimination, whereas the addition of pressure and venous oxygenation measurements would substantially improve classification.

## 2. Methods

### 2.1. Study Design and Two-Layer Modeling Strategy

This was a mechanistic computational study. The model had two deliberately separated layers. The hidden physiology layer generated a known cause of hypoxemia and solved the associated circulation. The observation layer converted that state into measurements that could plausibly be obtained in clinical care. Only observation-layer variables were provided to the machine learning models. Hidden PVR, pulmonary flow, systemic flow, oxygen consumption, collateral flow, pulmonary venous oxygenation, and ventricular pump parameters were never supplied to the classifier. This separation was intended to prevent information leakage and to make the classification question clinically interpretable. Importantly, the observation layer is not independent of the hidden physiology layer: bedside variables are downstream outputs of the same prespecified equations and phenotype perturbations used to generate the hidden states. The machine learning task should therefore be interpreted as an internal recoverability experiment—testing which encoded physiological states remain distinguishable after overlap and measurement noise—rather than as an estimate of diagnostic information or diagnostic accuracy in real patients. All simulations, statistical analyses, machine-learning models, and figure generation were performed in Python 3.13.5 using NumPy 2.3.5, pandas 2.2.3, scikit-learn 1.8.0, and Matplotlib 3.10.8. During the preparation of this manuscript and study, the authors used OpenAI ChatGPT (GPT-5.6 Sol; https://chatgpt.com/) as an assistive tool for selected computational and editorial tasks.

### 2.2. Closed-Loop Glenn Circulation

A steady-state zero-dimensional model represented a bidirectional Glenn without additional antegrade pulmonary blood flow. All flows were indexed to body surface area. The circuit contained an upper-body systemic pathway, the SVC/Glenn pathway, a pulmonary pathway, a lower-body systemic pathway, and the common atrial/systemic ventricular compartment. The model enforced conservation of flow, so pressure and flow could not be assigned independently. This pressure-flow framework follows prior analytical and patient-specific Glenn models [2,3,4,10].

Upper-body venous return was represented as Q_SVC = (MAP − P_SVC)/R_upper. Effective pulmonary flow was Q_p = (P_SVC − P_atrium)/R_p. Lower-body venous return was Q_IVC = (MAP − P_atrium)/R_lower. When no veno-venous collateral was present, Q_SVC = Q_p. When a VVC was present, Q_VVC = (P_SVC − P_atrium)/R_VVC and conservation required Q_SVC = Q_p + Q_VVC. Thus, increased pulmonary pathway resistance raised upstream SVC/Glenn pressure and altered effective pulmonary flow, whereas a VVC provided an alternative low-pressure pathway that reduced effective pulmonary participation and partially decompressed the SVC compartment [6,7].

### 2.3. Ventricular Pump Representation

To avoid defining ventricular dysfunction by hypotension, the systemic ventricle was represented as an effective pressure source with an internal pump resistance. Systemic arterial pressure was solved from MAP = P_atrium + P_source − R_vent × Q_s, where P_source represents the effective pressure head generated by the ventricle and R_vent represents internal pump resistance. Total systemic flow Q_s was the sum of Q_SVC and Q_IVC and therefore depended on MAP. The ventricular dysfunction phenotype incorporated both pump impairment and a compensatory vascular response. Effective ventricular pressure generation was reduced nonlinearly to 66–97% of the patient-specific baseline and internal pump resistance increased by approximately 3–58%. During mild and moderate impairment, upper- and lower-body systemic resistances increased progressively, reaching approximately 2.3 times baseline, representing compensatory vasoconstriction that could reduce systemic flow while preserving arterial pressure. In the most severe states, this compensatory increase was allowed to partially recede, permitting hypotension to emerge as a later feature. This staged response is a modeling approximation. Ref. [11] provides general postoperative evidence that blood pressure may remain preserved despite falling cardiac output. More directly relevant congenital and single-ventricle literature also emphasizes that conventional hemodynamic variables, including arterial pressure, incompletely reflect systemic oxygen transport and that venous oxygen saturation provides complementary information [13,14]. However, no cited study provides a quantitative Glenn-specific systemic-resistance compensation curve; the staged compensation used here therefore remains an explicit modeling assumption.

An earlier developmental formulation represented ventricular dysfunction primarily through reduced pressure-generating capacity without compensatory systemic vasoconstriction. During physiological review, this was recognized to make low MAP a prominent model-implied feature, and the compensated formulation was introduced. To disclose this model-development change transparently, we repeated the analysis in the final frozen framework while removing only the compensatory vasoconstriction rule. The noncompensated formulation yielded 67.5% accuracy, macro-AUROC 0.908, and ventricular dysfunction recall 85.4%, compared with 56.1%, 0.845, and 52.0%, respectively, in the primary compensated formulation. This comparison is reported as a model-dependence sensitivity analysis and demonstrates that classification performance depends materially on phenotype parameterization; it does not establish that either formulation has been clinically validated.

### 2.4. Numerical Solution and Physiologic Rejection Criteria

For each sampled virtual patient, the steady-state pressure-flow equations form a coupled linear system. The downstream Glenn resistance was represented as the pulmonary pathway resistance alone or as the parallel combination of pulmonary and VVC pathways when a collateral was present. The effective upper-body and lower-body conductances were then combined with the ventricular pressure-source equation, allowing MAP, SVC/Glenn pressure, Q_SVC, Q_p, Q_VVC, Q_IVC, and Q_s to be obtained algebraically in a single closed-form solution. No iterative optimizer was required. States with a singular or non-finite solution, nonpositive regional flow, or values outside the prespecified broad physiological bounds were rejected before cohort selection. Oxygen-content and saturation equations were solved only after a valid pressure-flow state had been established.

### 2.5. Oxygen Transport and Saturation

Blood oxygen content was approximated as CO_2_ = 1.34 × Hb × SO_2_; the dissolved oxygen component was omitted from the hidden oxygen-content balance because of its small contribution in the modeled saturation range. Total oxygen consumption was divided between upper- and lower-body circulations. Regional venous oxygen content was calculated from arterial oxygen content, regional blood flow, and regional oxygen use. At the common atrial/systemic ventricular mixing point, oxygen mass conservation required Q_s × C_aO2 = Q_p × C_pvO2 + Q_VVC × C_SVCO2 + Q_IVC × C_IVCO2. SpO_2_ and SVC saturation were therefore outputs of the solved circulation rather than assigned class labels.

### 2.6. Definition of Hidden Mechanisms

Five dominant mechanisms were specified before classification. Each changed a distinct hidden physiologic element while baseline patient characteristics continued to vary. The mechanisms and their expected bedside consequences are summarized in Table 1.

### 2.7. Virtual-Patient Generation and Parameterization

Virtual patients were generated by Monte Carlo sampling from prespecified baseline distributions. Hemoglobin was centered at 15.0 g/dL (SD 1.4), common atrial pressure at 5 mmHg (SD 1.0), effective pulmonary pathway resistance at 3.0 WU.m^2^ (SD 0.65), oxygen consumption at 160 mL/min/m^2^ (SD 24), ventricular pressure source at 68 mmHg (SD 6.5), and ventricular internal resistance at 1.8 mmHg·min·m^2^/L (SD 0.35). Upper-body and lower-body effective systemic resistances were centered at 28 and 32 mmHg·min·m^2^/L, respectively, and varied between patients. Because flow was indexed to body surface area, resistance calculated as pressure divided by indexed flow is expressed as mmHg·min·m^2^/L. The pressure, pulmonary flow, PVR, and metabolic scale were anchored to published Glenn CMR, catheterization, and modeling studies [2,3,4,9,10]. Values not directly available as Glenn-specific distributions, including pump, compensatory vascular, and heart-rate response parameters, were treated explicitly as modeling assumptions and examined through ablation or sensitivity analyses.

The pulmonary gas-exchange phenotype used a hidden gas-exchange efficiency parameter that reduced pulmonary venous oxygenation without changing the underlying cavopulmonary resistance. The purpose was to represent impaired oxygen transfer while keeping the mechanism distinct from pulmonary vascular flow limitation. Because the model does not contain a full ventilation-perfusion or ventilator-response model, FiO_2_, PaO_2_, PaCO_2_, and pH were not used as classification features in the primary analysis. These standard deviations were simulation assumptions and were not intended to represent validated total clinical error for catheter-derived measurements, which may additionally vary with transducer leveling, catheter position, sampling, loading conditions, and temporal mismatch.

### 2.8. Generation of Bedside-Observable Markers

After the hidden state was solved, the observation layer generated SpO_2_, MAP, Glenn/SVC pressure, SVC saturation, hemoglobin, and heart rate. Gaussian observation error was added to SpO_2_ (SD 1.2 percentage points), Glenn/SVC pressure (1 mmHg), MAP (2 mmHg), hemoglobin (0.25 g/dL), SVC saturation (1.8 percentage points), and heart rate (3 beats/min). These were simulation assumptions rather than validated estimates of total clinical measurement variability. Reviewer-requested stress tests therefore included twofold and threefold inflation of all observation-error SDs and a targeted high-catheter-variability scenario using Glenn/SVC pressure SD 3 mmHg and SVC saturation SD 5 percentage points while retaining baseline error for other variables.

Heart rate was modeled as a secondary physiologic response rather than a hidden class label. A patient-specific baseline heart rate (mean 125 beats/min, SD 14) was increased according to excess metabolic demand and the degree of systemic hypotension, with additional measurement variability. Because this relation is a modeling assumption rather than a Glenn-specific validated equation, its importance was tested by removing heart rate from the classifier; the principal results were unchanged.

Respiratory variables were deliberately excluded from the primary machine learning analysis. In preliminary development, FiO_2_ and PaCO_2_ distributions were partly linked to the simulated pulmonary gas-exchange phenotype, and PaO_2_ was derived from the modeled arterial saturation. Using those variables for classification would therefore risk feature-definition leakage and would overstate their independent bedside information. Carbon dioxide can also directly alter Glenn flow and oxygenation [15]. A future clinical validation study should evaluate independently measured blood gases and respiratory support variables rather than synthetic respiratory surrogates.

NIRS was retained only as a secondary sensitivity feature because it was synthetically derived primarily from venous oxygenation. Lactate was removed from the main analysis: delayed production and clearance cannot be adequately represented by this steady-state model.

Equal class size was used to compare recoverability across the five encoded states, but it does not represent clinical prevalence and therefore cannot support clinical positive or negative predictive values. Real Glenn hypoxemia may also involve more than one mechanism simultaneously; the single-dominant-mechanism task is an intentional simplification rather than a representation of clinical prevalence or complexity.

### 2.9. Cohort Selection and Prevention of Trivial Classification

A total of 150,000 candidate states were generated (30,000 per mechanism) before physiological filtering. Broad validity filters required finite solutions, pulmonary flow 0.3–5.0 L/min/m^2^, systemic flow 1.0–8.0 L/min/m^2^, Glenn/SVC pressure 3–30 mmHg, MAP 25–100 mmHg, SpO_2_ 40–95%, and SVC saturation 20–90%; 127,162 states remained. The strict cohort then required observed SpO_2_ 65–80%, internal Qp 0.8–3.2 L/min/m^2^, internal Qs 1.8–6.0 L/min/m^2^, and Glenn/SVC pressure 5–24 mmHg; 66,269 states met these criteria. Qp and Qs were hidden plausibility filters and were never exposed to the classifier.

Because the five simulated mechanisms had different acceptance rates after filtering, the primary dataset was down-sampled to the smallest class. The balanced cohort contained 26,400 patients (5280 per mechanism), with 19,800 for training and 6600 for held-out testing. Equal class size was an experimental design choice and does not represent clinical prevalence; clinical predictive values therefore cannot be inferred. The full unbalanced strict cohort was also analyzed as a reviewer-requested sensitivity analysis. A fixed random seed (20260812) was used.

### 2.10. Machine Learning Models and Statistical Analysis

Three prespecified information levels were evaluated. Model 1 used SpO_2_ alone. Model 2 represented noninvasive bedside information and used SpO_2_, heart rate, MAP, and hemoglobin. Model 3, the primary Glenn bedside model, added Glenn/SVC pressure and SVC saturation. A secondary NIRS-augmented model tested whether synthetic NIRS added information to Model 3. Hidden PVR, Qp, Qs, oxygen consumption, VVC flow, pulmonary gas-exchange efficiency, ventricular pump parameters, and synthetic respiratory variables were never supplied to the primary classifier.

The primary classifier was multinomial logistic regression after standardization using training-set parameters. A 500-tree class-weighted random forest served as a nonlinear comparator. Performance was assessed in the held-out test set using five-class accuracy and macro-averaged one-versus-rest AUROC. For the primary model, 1000 bootstrap resamples provided 95% confidence intervals for accuracy, macro-AUROC, and mechanism-specific recall. A normalized confusion matrix characterized misclassification, and leave-one-variable-out ablation quantified incremental information. Exploratory internal probability calibration was summarized by the multiclass Brier score and a 10-bin top-label expected calibration error; these assess self-consistency within synthetic labels, not clinical calibration.

Sensitivity analyses independently regenerated the virtual population after twofold and threefold inflation of observation error; a targeted high-catheter-variability scenario (Glenn/SVC pressure SD 3 mmHg; SVC saturation SD 5 percentage points); hemoglobin shifts of −2 or +2 g/dL; baseline pulmonary resistance changes of −20% or +25%; and baseline oxygen consumption changes of −15% or +20%. The same filters were applied, and each five-class cohort was independently rebalanced. Generated, broad-valid, strict-cohort, and final balanced sample sizes were recorded for every scenario because changes in filter acceptance alter cohort composition. A controlled model-dependence analysis also removed compensatory systemic vasoconstriction from the ventricular phenotype while retaining the other specifications.

### 2.11. Additional Physiologic Robustness Analyses

Three additional analyses were performed in response to reviewer concerns, with the primary multinomial logistic-regression classifier frozen and not retrained. First, downstream filling pressure was examined within the ventricular dysfunction phenotype by increasing the hidden common atrial pressure progressively with ventricular dysfunction severity. Two stress-test magnitudes were evaluated: a maximum severity-dependent increase of +3 mmHg and +6 mmHg above the patient-specific baseline atrial pressure. Thirty thousand candidate ventricular dysfunction states were generated for each scenario and passed through the same broad and strict physiological filters. Atrial pressure remained hidden from the classifier. We recorded median Glenn/SVC pressure and the proportions classified as ventricular dysfunction or elevated PVR.

Second, a targeted mixed-mechanism stress test simultaneously applied elevated PVR (the same 1.5–3.2-fold pulmonary resistance perturbation used in the primary elevated-PVR phenotype) and pulmonary gas-exchange impairment (the same reduction in hidden pulmonary venous oxygenation used in the primary gas-exchange phenotype), with independent severity draws. Thirty thousand candidate states were generated and filtered using the same criteria. Because the fixed classifier was trained on mutually exclusive single-mechanism labels, no conventional accuracy was assigned to this mixed state. Instead, we reported the proportion for which either constituent mechanism was the top-ranked class and the proportion for which both constituent mechanisms appeared among the two highest predicted probabilities.

Third, the primary classifier was compared with a transparent physiological decision rule using the same held-out test cohort. Rule thresholds were derived only from class-specific medians in the training set, with no test-set tuning. The resulting rule assigned elevated PVR when Glenn/SVC pressure was at least 14.18 mmHg; among the remaining states, pulmonary gas-exchange impairment was assigned when SpO_2_ was at most 69.19% with SVC saturation above 54.36%; among states with SVC saturation at or below 54.36%, increased metabolic demand was assigned when heart rate was at least 128.33 beats/min and MAP at least 66.10 mmHg, otherwise ventricular dysfunction was assigned; remaining states were assigned to VVC shunting. Overall accuracy and mechanism-specific recall were compared with multinomial logistic regression on the identical held-out observations.

### 2.12. Reference-State Physiological Plausibility Check

Before phenotype generation, an unperturbed reference population was simulated using the same baseline distributions but without a disease-specific perturbation. This was a descriptive plausibility check rather than patient-level external validation. Model-generated pulmonary flow and Glenn/SVC pressure were compared with the scale of published CMR and catheterization measurements [3,4].

## 3. Results

### 3.1. Cohort Generation and Bedside-Marker Distributions

Of 150,000 candidate states generated in total, 127,162 passed broad physiological validity filters, and 66,269 met the strict hypoxemia/hemodynamic criteria. After balancing, 26,400 virtual patients were analyzed (5280 per mechanism); the training set contained 19,800 observations and the held-out test set 6600.

### 3.2. Reference-State Physiological Plausibility

In 25,000 unperturbed reference simulations, the physiologically valid population produced mean effective pulmonary flow of 2.01 +/− 0.34 L/min/m^2^ and mean Glenn/SVC pressure of 11.0 +/− 1.8 mmHg. The pulmonary flow value was similar to the summed CMR LPA and RPA flow reported by Laudenschlager et al. (0.971 + 0.998 = 1.97 L/min/m^2^) [3], and the modeled Glenn pressure was within the low-teen SVC pressure range reported across contemporary multicenter Glenn catheterization data [4]. This check supports the scale of the unperturbed circulation but is not patient-level validation.

### 3.3. Bedside-Marker Distributions

SpO_2_ intentionally overlapped across mechanisms, with median values between 68.8% and 72.0% (Table 2). Elevated PVR showed the highest Glenn/SVC pressure, whereas increased metabolic demand showed the lowest SVC saturation. In the compensated ventricular phenotype, arterial pressure overlapped substantially with the other mechanisms despite reduced hidden systemic flow. The 95% simulation intervals overlapped across several bedside variables, emphasizing that no single marker uniquely identified an encoded mechanism.

Within the ventricular dysfunction phenotype, the model reproduced a compensated low-output phase. Median MAP was 64.7 mmHg in mild states and 64.3 mmHg in moderate states, while median systemic flow fell from 3.10 to 2.20 L/min/m^2^. In severe states, median MAP decreased to 51.2 mmHg as systemic flow remained low at 2.06 L/min/m^2^. SVC saturation fell, and heart rate increased across this progression. Thus, hypotension emerged principally after compensatory vascular responses became insufficient rather than serving as the initial marker of reduced systemic flow.

### 3.4. Classification Using Progressively Available Bedside Information

SpO_2_ alone provided limited mechanistic discrimination (26.1% accuracy; macro-AUROC 0.580). The noninvasive bedside model achieved 30.3% accuracy and macro-AUROC 0.632. The primary Glenn bedside model achieved 56.1% accuracy (95% CI 55.0–57.4%) and macro-AUROC 0.845 (95% CI 0.840–0.851). Adding synthetic NIRS did not materially change performance (56.0%; macro-AUROC 0.845).

### 3.5. Mechanism-Specific Performance and Variable Contribution 

**Figure 1:**
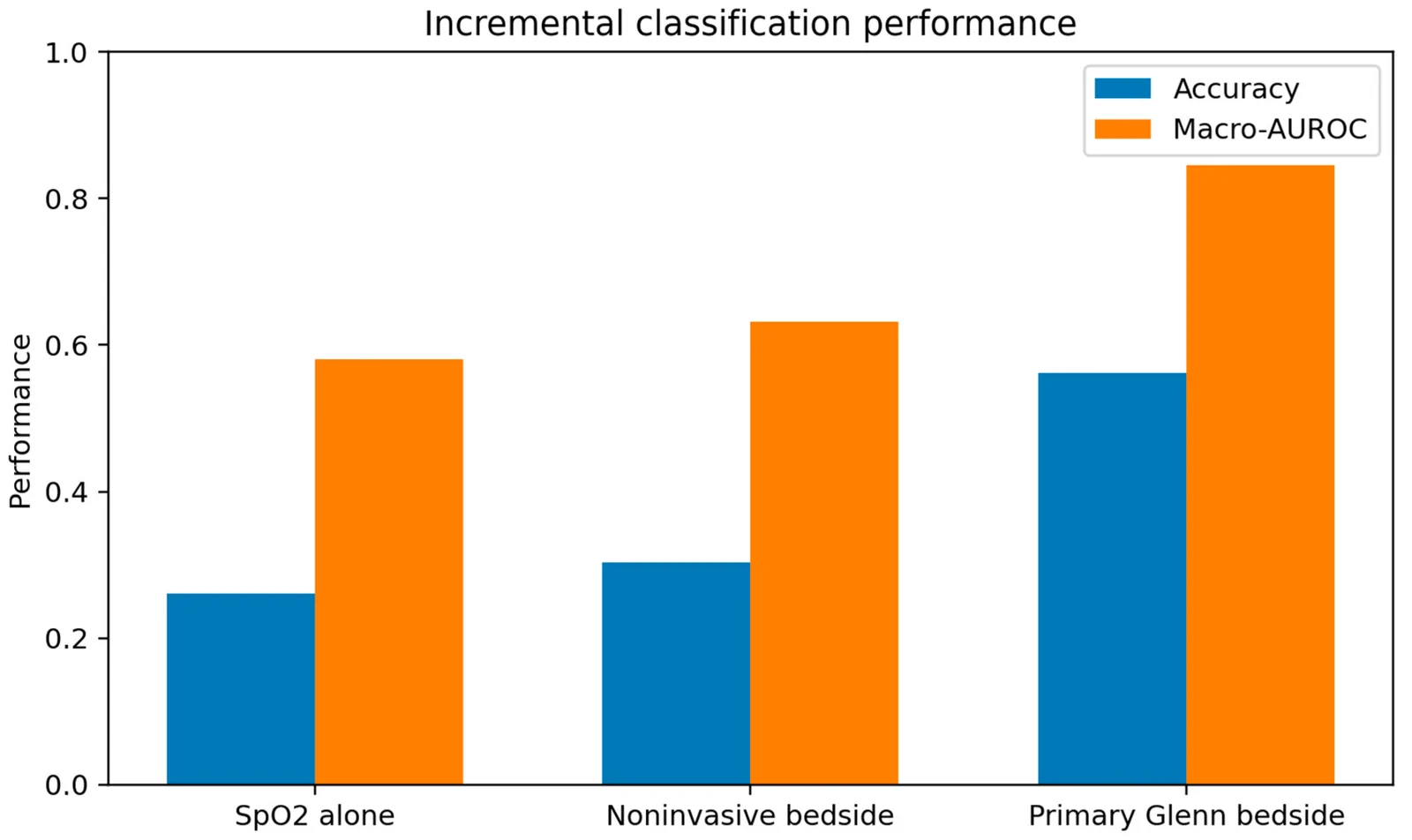
Incremental classification performance using bedside-observable markers. Values quantify internal recoverability within the specified mechanistic model and should not be interpreted as clinical diagnostic performance. Abbreviations: SpO_2_, peripheral oxygen saturation; macro-AUROC, macro-averaged area under the receiver operating characteristic curve.

**Figure 2:**
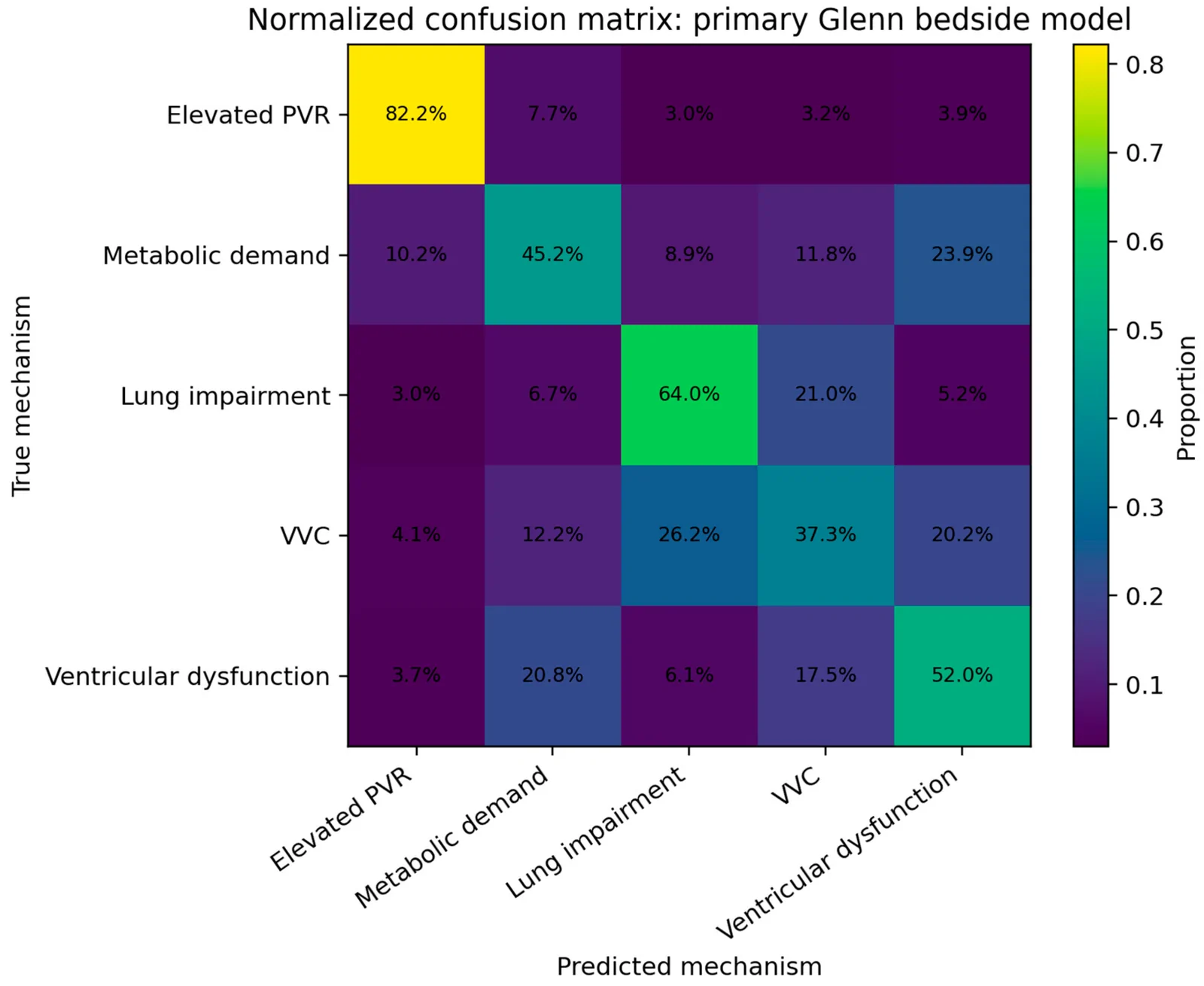
Normalized confusion matrix for the final primary Glenn bedside model. Residual overlap reflects model-implied ambiguity among encoded physiologic states. Abbreviations: PVR, pulmonary vascular resistance; VVC, veno-venous collateral.

In the primary bedside model, recall was 82.2% for elevated PVR (95% CI 80.3–84.3%), 64.0% for pulmonary gas-exchange impairment (95% CI 61.4–66.5%), 45.2% for increased metabolic demand (95% CI 42.3–47.9%), 52.0% for ventricular dysfunction (95% CI 49.3–54.7%), and 37.3% for VVC shunting (95% CI 34.7–40.0%). Residual ambiguity involved VVC shunting versus pulmonary gas-exchange impairment and compensated ventricular dysfunction versus metabolic/VVC patterns. These results are summarized in Figure 1 and Figure 2 and Table 3.

Ablation confirmed that Glenn/SVC pressure and SVC saturation carried the largest incremental information. Removing Glenn/SVC pressure reduced accuracy from 56.1% to 41.7% (macro-AUROC 0.744); removing SVC saturation reduced accuracy to 46.1% (AUROC 0.765); and removing SpO_2_ reduced accuracy to 47.0% (AUROC 0.776). Removing MAP, heart rate, or hemoglobin produced comparatively small changes (54.5%, 55.9%, and 55.8% accuracy, respectively).

### 3.6. Sensitivity Analyses

Sensitivity analyses showed substantial dependence on observation quality. The independently regenerated baseline reproducibility run achieved 57.3% accuracy and macro-AUROC 0.854; twofold measurement noise reduced performance to 47.6%/0.782, threefold noise to 39.4%/0.715, and the targeted high-catheter-variability scenario to 45.6%/0.762. Across all sensitivity scenarios, final sample sizes differed because fixed filters selected different proportions of generated states; complete cohort flow is provided in Supplementary Table S1.

### 3.7. Model Dependence, Class Composition, and Internal Calibration

Removing compensatory systemic vasoconstriction from the ventricular phenotype increased accuracy from 56.1% to 67.5%, macro-AUROC from 0.845 to 0.908, and ventricular dysfunction recall from 52.0% to 85.4%. This demonstrates material dependence on phenotype parameterization. In the full unbalanced strict cohort (N = 66,269), accuracy was 64.7% and macro-AUROC 0.870, while mechanism-specific recall ranged from 12.4% to 88.0%, demonstrating dependence on simulated class composition. Exploratory internal calibration yielded a multiclass Brier score of 0.546 and 10-bin top-label expected calibration error of 0.014; these do not constitute clinical calibration.

### 3.8. Downstream Atrial Pressure, Mixed Mechanisms, and Physiological-Rule Comparison

Progressively increasing hidden atrial pressure during ventricular dysfunction increased overlap with the elevated-PVR phenotype. With no imposed atrial pressure rise, the ventricular dysfunction stress-test cohort had a median Glenn/SVC pressure of 9.80 mmHg; 50.7% of states were classified as ventricular dysfunction and 4.1% as elevated PVR. With a maximum severity-dependent atrial pressure rise of +3 mmHg, median Glenn/SVC pressure increased to 10.29 mmHg, ventricular dysfunction attribution decreased to 43.7%, and elevated-PVR attribution increased to 5.4%. With a maximum rise of +6 mmHg, median Glenn/SVC pressure increased to 10.75 mmHg, ventricular dysfunction attribution decreased to 36.4%, and elevated-PVR attribution increased to 7.3%. These results confirm that downstream filling pressure can erode the specificity of Glenn pressure for pulmonary vascular resistance.

In the targeted mixed elevated PVR plus gas-exchange impairment stress test, 5553 states met the strict cohort criteria. The fixed single-label classifier assigned 63.4% to elevated PVR and 30.5% to pulmonary gas-exchange impairment; either constituent mechanism was the top-ranked class in 93.9% of states, while both constituent mechanisms appeared among the two highest predicted probabilities in 55.2%. These values describe attribution behavior of a single-label classifier in a mixed state and are not measures of diagnostic accuracy.

The transparent physiological decision rule achieved 44.6% accuracy in the same held-out test cohort, compared with 56.1% for multinomial logistic regression. Rule-based recall was 77.4% for elevated PVR, 57.4% for VVC shunting, 15.8% for pulmonary gas-exchange impairment, 19.8% for increased metabolic demand, and 52.7% for ventricular dysfunction. Thus, within the same simulated framework, multivariable logistic regression provided an 11.5-percentage-point improvement in overall accuracy over the simple threshold rule, driven particularly by improved separation of pulmonary gas-exchange impairment and increased metabolic demand. Full results are provided in Supplementary Table S2.

## 4. Discussion

This study examined whether routinely available bedside markers could recover major physiological states encoded in a bidirectional Glenn simulation. SpO_2_ alone provided limited discrimination. The largest incremental information came from Glenn/SVC pressure and SVC saturation, whereas heart rate and MAP contributed comparatively little after the other variables were included. These findings represent internal mechanistic recoverability rather than clinical diagnostic performance. Because the hidden phenotypes were created through prespecified physiological perturbations that themselves generate the observed bedside variables, the classifier necessarily detects, in part, structure imposed by the model. The value of the analysis is therefore not the absolute accuracy or AUROC, but the identification of which encoded states remain distinguishable after physiological overlap and measurement uncertainty and which remain difficult to separate.

Glenn/SVC pressure provided the largest incremental information. This is physiologically consistent with a passive pulmonary circulation in which flow depends on upstream venous pressure, downstream atrial pressure, and pulmonary resistance [2,3,4]. In clinical reports, pulmonary vasodilation with inhaled nitric oxide after Glenn decreased central venous pressure while improving arterial and venous oxygen saturation [5]. The model therefore predicts that, among patients with comparable hypoxemia, a higher Glenn pressure should increase the probability of pulmonary vascular or mechanical flow limitation; it is not proposed as a specific diagnostic rule.

VVC shunting remained difficult to separate from pulmonary gas-exchange impairment using the primary hemodynamic markers. This is physiologically plausible: both may cause cyanosis without marked Glenn pressure elevation. VVCs are recognized decompressive venous pathways after cavopulmonary connection [6,7], whereas pulmonary gas-exchange changes can alter saturation without a proportional change in Qp/Qs [8]. We did not use synthetic blood-gas or FiO_2_ variables to improve this distinction because those variables were partly generated from the lung-impairment definition and would risk information leakage. In clinical validation, independently measured respiratory support and blood-gas data should be tested prospectively.

The ventricular dysfunction phenotype illustrates both a clinically important concept and the dependence of classification on modeling choices. In the primary formulation, reduced pump performance was coupled to compensatory systemic vasoconstriction, allowing systemic flow and venous oxygenation to deteriorate while MAP remained relatively preserved during mild and moderate impairment. This concept is supported qualitatively by postoperative congenital heart and single-ventricle literature [11,13,14], but no Glenn-specific study defines the quantitative compensation curve used here. In the controlled final sensitivity analysis, removing compensatory vasoconstriction increased overall accuracy from 56.1% to 67.5%, macro-AUROC from 0.845 to 0.908, and ventricular dysfunction recall from 52.0% to 85.4%. This large change is reported specifically to demonstrate parameterization dependence rather than to favor one formulation as clinically validated.

The downstream atrial pressure sensitivity analysis directly addresses an additional limitation of the primary ventricular phenotype. Increasing hidden atrial pressure progressively raised Glenn/SVC pressure and shifted classification away from ventricular dysfunction toward elevated PVR. This behavior is physiologically expected because Glenn flow depends on the pressure gradient between the SVC/Glenn pathway and the downstream atrial compartment. The result reinforces that an elevated Glenn pressure is not specific for increased PVR when downstream filling pressure is also elevated and that atrial or ventricular filling-pressure information should be incorporated in future clinical validation.

The mixed PVR plus gas-exchange analysis further illustrates the limitation of a mutually exclusive single-label target. Most mixed states were attributed to one of their two constituent mechanisms, but both mechanisms appeared among the two highest predicted probabilities in only approximately half of states. The current classifier therefore should not be interpreted as identifying all simultaneous causes of hypoxemia. Rather, the mixed-phenotype stress test shows how the encoded bedside signature is partitioned when two mechanisms coexist.

Comparison with a simple physiological decision rule also helps define what the statistical classifier adds within the simulation. The rule achieved 44.6% accuracy versus 56.1% for multinomial logistic regression on the identical held-out cohort. The difference suggests that multivariable weighting provides additional separation beyond a small set of intuitive thresholds, particularly for gas-exchange impairment and increased metabolic demand. However, both approaches operate entirely within the same model-generated data and this comparison does not establish superiority in clinical patients.

Increased metabolic demand preferentially lowered SVC saturation and increased the modeled heart rate response, consistent with the expected effect of higher extraction on venous oxygenation [2,9]. Heart rate contributed modestly to overall discrimination, while removal of MAP had little effect after compensated ventricular dysfunction was introduced. These findings emphasize that normal arterial pressure does not exclude impaired systemic output in the modeled Glenn circulation.

NIRS was intentionally kept secondary because it was generated largely from venous oxygenation and therefore added little independent information once SVC saturation was available. Lactate was removed from the main analysis because a steady-state model cannot reproduce delayed lactate production and clearance. These choices favor a parsimonious feature set over adding synthetic markers simply because they are clinically measurable.

Several limitations are central to interpretation. First, structural circularity is inherent to this simulation: each hidden phenotype is created by prespecified perturbations that, through the same equations, generate the bedside variables later used by the classifier. Performance therefore measures recoverability of model-encoded states under selected assumptions and does not independently establish bedside information content in patients. Second, the cohort is synthetic and class-balanced; simulated prevalence is artificial and clinical predictive values cannot be inferred. The unbalanced strict cohort analysis showed marked variation in mechanism-specific recall, confirming dependence on simulated class composition. Third, the primary task assigns a single dominant mechanism, whereas real Glenn hypoxemia may be multifactorial. The targeted combined elevated-PVR plus gas-exchange impairment analysis demonstrates this limitation but does not represent the broader range of simultaneous physiological disturbances that occur clinically. Fourth, the primary ventricular phenotype does not dynamically generate filling-pressure elevation from a pressure-volume model; the reviewer-requested imposed atrial pressure stress test showed that higher downstream pressure increases overlap with elevated PVR, but it remains a simplified sensitivity analysis rather than validated ventricular-atrial coupling. Fifth, internal probability calibration against synthetic labels does not establish clinical calibration. Sixth, observation-error assumptions were not derived from a Glenn-specific measurement study; performance deteriorated substantially under twofold, threefold, and high-catheter-variability stress tests. Additional limitations include the steady-state zero-dimensional representation, model-assumed response coefficients including systemic vascular compensation, simplified gas-exchange physiology, exclusion of antegrade pulmonary blood flow and several other sources of cyanosis [8,10,16,17], and incomplete availability of Glenn pressure and SVC saturation in clinical care.

The next step is clinical validation using synchronized bedside data linked to independent mechanistic adjudication from catheterization, echocardiography, cross-sectional imaging, respiratory assessment, and clinical course. Such validation should include direct or surrogate assessment of downstream atrial/ventricular filling pressure and should permit more than one physiological mechanism to coexist. The model generates specific hypotheses for that validation: elevated Glenn pressure should increase the likelihood of pulmonary vascular or mechanical flow limitation but may also reflect elevated downstream filling pressure; VVC and pulmonary gas-exchange abnormalities may require respiratory and imaging context for separation; disproportionate venous desaturation should accompany increased extraction; and reduced systemic output may initially present with preserved MAP, lower venous oxygen saturation, and compensatory tachycardia before hypotension develops.

In conclusion, this mechanistic simulation shows that some prespecified Glenn physiological states remain partially recoverable from their model-generated bedside signatures after overlap and observation noise are introduced. Glenn/SVC pressure and SVC saturation contributed the greatest incremental discrimination, whereas SpO_2_ alone and MAP alone were less informative in the primary compensated formulation. Multinomial logistic regression outperformed a simple physiological threshold rule within the same simulated test cohort (56.1% versus 44.6% accuracy), indicating additional value from multivariable weighting within the model. However, elevated downstream atrial pressure increased overlap between ventricular dysfunction and elevated PVR, and the mixed PVR plus gas-exchange stress test demonstrated the limitations of a mutually exclusive single-label classifier. The numerical performance estimates are materially dependent on phenotype definitions, parameter distributions, class composition, and measurement assumptions and should not be interpreted as clinical diagnostic accuracy or as independent evidence that the same relationships occur in patients. Their principal value is to define explicit, testable hypotheses and areas of expected overlap for prospective validation using independently measured clinical Glenn data.

## Figures and Tables

**Table 1:** Hidden physiologic mechanisms used to generate the virtual cohort.

Mechanism	Internal Perturbation (Hidden)	Model-Implied Bedside Consequence	Physiologic Support
Elevated PVR	Increase effective pulmonary pathway resistance 1.5–3.2×; preserve intrinsic pulmonary gas exchange	Higher Glenn/SVC pressure with hypoxemia; venous oxygenation may fall	Passive Glenn pressure-flow/PVR studies [3,4]; nitric oxide response [5]
VVC shunting	Add graded venous bypass from SVC toward a lower-pressure venous/atrial compartment	Hypoxemia with relatively lower/decompressed Glenn pressure than isolated high PVR	Systemic venous collateral series [6,7]
Pulmonary gas-exchange impairment	Reduce hidden pulmonary gas-exchange efficiency while preserving cavopulmonary resistance	Low SpO_2_ without obligatory Glenn-pressure elevation; clinical separation may require independent respiratory data	Oxygen can raise saturation without material Qp/Qs change [8]
Increased metabolic demand	Increase regional oxygen consumption 1.2–1.8× baseline	Lower SVC saturation and compensatory higher heart rate with relatively preserved Glenn pressure	Flow-metabolism/oxygen-consumption studies [2,9]
Ventricular dysfunction	Reduce ventricular pressure generation and increase internal pump resistance; increase systemic resistance during mild/moderate impairment with partial loss of compensation in severe states	Reduced systemic flow with initially preserved MAP, lower venous oxygenation, and compensatory tachycardia; hypotension emerges later with decompensation	Flow and oxygen-delivery framework [10]; preserved blood pressure despite falling postoperative cardiac output [11]

Because flows were indexed to body surface area, resistance was expressed as pressure divided by indexed flow (mmHg·min·m^2^/L), dimensionally equivalent to indexed Wood units. Abbreviations: PVR, pulmonary vascular resistance; VVC, veno-venous collateral; SVC, superior vena cava.

**Table 2:** Bedside-marker distributions in the strict hypoxemic cohort. Values are median (2.5th–97.5th percentile).

Mechanism	SpO_2_ (%)	HR (bpm)	MAP (mmHg)	Glenn/SVC (mmHg)	SVC Sat (%)	Hb (g/dL)
Elevated PVR	72.0 (65.4–79.2)	125.9 (98.1–155.1)	66.6 (55.3–78.5)	17.0 (10.8–23.2)	54.6 (42.4–67.5)	15.1 (12.5–17.9)
VVC shunting	71.6 (65.4–79.1)	125.9 (97.5–154.2)	66.9 (55.4–78.2)	10.5 (6.8–14.4)	56.7 (45.7–69.0)	15.2 (12.6–17.9)
Pulmonary gas-exchange impairment	68.8 (65.2–76.7)	126.2 (97.8–154.4)	67.6 (56.2–78.9)	11.3 (7.5–15.6)	55.3 (46.5–67.1)	15.4 (12.7–18.1)
Increased metabolic demand	69.6 (65.2–78.3)	130.1 (101.7–159.1)	67.7 (56.1–78.9)	11.2 (7.4–15.6)	50.6 (41.0–64.9)	15.4 (12.8–18.1)
Ventricular dysfunction	70.3 (65.3–78.8)	126.1 (97.9–154.4)	64.3 (53.2–75.5)	9.8 (6.3–13.8)	52.1 (41.4–66.6)	15.4 (12.6–18.1)

Abbreviations: HR, heart rate; MAP, mean arterial pressure; SVC, superior vena cava; PVR, pulmonary vascular resistance; VVC, veno-venous collateral; SpO_2_, peripheral oxygen saturation; Hb, hemoglobin.

**Table 3:** Machine learning performance, uncertainty, variable contribution, and sensitivity analyses.

Analysis	Specification	Accuracy/Recall	95% CI	Macro-AUROC
Panel A	Model performance			
SpO_2_ alone	SpO_2_	0.261		0.580
Noninvasive bedside	SpO_2_ + HR + MAP + Hb	0.303		0.632
Primary Glenn bedside	+Glenn/SVC pressure + SVC saturation	0.561	0.550–0.574	0.845 (0.840–0.851)
NIRS augmented	Primary + synthetic NIRS	0.560		0.845
Random forest comparator	500 trees; primary inputs	0.542		0.831
Panel B	Mechanism-specific recall			
Elevated PVR		0.822	0.803–0.843	
VVC shunting		0.373	0.347–0.400	
Pulmonary gas-exchange impairment		0.640	0.614–0.665	
Increased metabolic demand		0.452	0.423–0.479	
Ventricular dysfunction		0.520	0.493–0.547	
Panel C	Leave-one-variable-out ablation			
Remove Glenn pressure	Primary minus one marker	0.417		0.744
Remove SVC saturation	Primary minus one marker	0.461		0.765
Remove SpO_2_	Primary minus one marker	0.470		0.776
Remove MAP	Primary minus one marker	0.545		0.838
Remove HR	Primary minus one marker	0.559		0.844
Remove Hb	Primary minus one marker	0.558		0.842
Panel D	Sensitivity analyses			
Baseline reproducibility	strict N = 66,191; balanced N = 26,270	0.573		0.854
2× measurement noise	strict N = 63,841; balanced N = 24,910	0.476		0.782
3× measurement noise	strict N = 60,218; balanced N = 23,265	0.394		0.715
High catheter variability	strict N = 63,267; balanced N = 24,505	0.456		0.762
Hb −2 g/dL	strict N = 44,553; balanced N = 16,190	0.548		0.837
Hb +2 g/dL	strict N = 81,767; balanced N = 36,025	0.594		0.863
Baseline PVR −20%	strict N = 70,973; balanced N = 27,630	0.560		0.843
Baseline PVR +25%	strict N = 58,380; balanced N = 24,655	0.560		0.853
VO_2_ −15%	strict N = 83,897; balanced N = 37,665	0.584		0.859
VO_2_ +20%	strict N = 38,500; balanced N = 13,895	0.548		0.836
Panel E	Model-dependence/class composition			
Noncompensated ventricular formulation	balanced N = 46,025	0.675		0.908
Unbalanced strict cohort	N = 66,269	0.647		0.870

Abbreviations: PVR, pulmonary vascular resistance; VVC, veno-venous collateral; SVC, superior vena cava; CI, confidence interval; NIRS, near-infrared spectroscopy; macro-AUROC, macro-averaged area under the receiver operating characteristic curve; Hb, hemoglobin; MAP, mean arterial pressure; SpO_2_, peripheral oxygen saturation; HR, heart rate; VO_2_, oxygen consumption; N, sample size.

## Data Availability

No patient-level or externally sourced clinical data were used in this study. All data analyzed were synthetically generated from the computational model described in the Methods. The simulation code, machine learning analysis code, parameter definitions, synthetic datasets, sensitivity analyses, and supplementary reproducibility materials are publicly available at: https://github.com/savorgnan5/glenn-hypoxemia-mechanistic-ml (accessed on 1 October 2026).

## Supplementary Materials

The following supporting information can be downloaded at: https://www.mdpi.com/article/10.3390/children13101414/s1, Table S1: Cohort flow and classification performance across sensitivity-analysis scenarios; Table S2: Reviewer-requested robustness analyses: physiologic-rule comparison, atrial-pressure sensitivity, and mixed PVR plus gas-exchange impairment.
