# Supplementary material for: Machine Learning to Distinguish Physiologic Causes of Hypoxemia After Bidirectional Glenn Using Bedside Markers
Source: Children (Basel). 2026 Oct 15;13(10):1414. doi: 10.3390/children13101414 (PMC13703036; doi:10.3390/children13101414)
Supplement: Supplementary file 1 [file children-13-01414-s001.zip › children-4579671-supplementary.pdf]

Supplementary Tables

Table S1. Cohort flow and classification performance across sensitivity-analysis scenarios

Scenario	Generated	Broad-valid	Strict cohort	Smallest class	Balanced N	Accuracy	Macro-AUROC
Baseline reproducibility	150000	127235	66191	5254	26270	0.573	0.854
2x measurement noise	150000	126598	63841	4982	24910	0.476	0.782
3x measurement noise	150000	124744	60218	4653	23265	0.394	0.715
High catheter variability	150000	124892	63267	4901	24505	0.456	0.762
Hb -2 g/dL	150000	114445	44553	3238	16190	0.548	0.837
Hb +2 g/dL	150000	134470	81767	7205	36025	0.594	0.863
Baseline PVR -20%	150000	128303	70973	5526	27630	0.560	0.843
Baseline PVR +25%	150000	124957	58380	4931	24655	0.560	0.853
VO2 -15%	150000	136121	83897	7533	37665	0.584	0.859
VO2 +20%	150000	110350	38500	2779	13895	0.548	0.836

Table S2. Reviewer-requested robustness analyses: physiologic-rule comparison, atrial-pressure sensitivity, and mixed PVR plus gas-exchange impairment

Analysis	Metric	Value/N	Median Glenn/SVC pressure (mmHg)	Additional result
Physiologic rule comparator	Logistic regression accuracy	0.561		
Physiologic rule comparator	Simple physiologic rule accuracy	0.446		
Physiologic rule comparator	Rule recall: Elevated PVR	0.774		
Physiologic rule comparator	Rule recall: VVC shunting	0.574		
Physiologic rule comparator	Rule recall: Pulmonary gas-exchange impairment	0.158		
Physiologic rule comparator	Rule recall: Increased metabolic demand	0.198		
Physiologic rule comparator	Rule recall: Ventricular dysfunction	0.527		
Atrial-pressure sensitivity	Maximum severity-dependent atrial-pressure increase +0 mmHg	5285	9.80	VD predicted 50.7%; PVR predicted 4.1%
Atrial-pressure sensitivity	Maximum severity-dependent atrial-pressure increase +3 mmHg	5297	10.29	VD predicted 43.7%; PVR predicted 5.4%
Atrial-pressure sensitivity	Maximum severity-dependent atrial-pressure increase +6 mmHg	5301	10.75	VD predicted 36.4%; PVR predicted 7.3%
Mixed PVR + gas exchange	Strict cohort N	5553	16.99	Top-1 either component 93.9%; both components top-2 55.2%
Mixed PVR + gas exchange	Predicted class: Elevated PVR	63.4		percent
Mixed PVR + gas exchange	Predicted class: VVC shunting	1.8		percent
Mixed PVR + gas exchange	Predicted class: Pulmonary gas-exchange impairment	30.5		percent
Mixed PVR + gas exchange	Predicted class: Increased metabolic demand	3.8		percent
Mixed PVR + gas exchange	Predicted class: Ventricular dysfunction	0.5		percent
